# Supplementary material for: Nonclassical mechanisms to irreversibly suppress β-hematin crystal growth
Source: Commun Biol. 2023 Jul 27;6:783. doi: 10.1038/s42003-023-05046-z (PMC10374632; doi:10.1038/s42003-023-05046-z)
Supplement: Supplementary file 2 — Supplemental Figures [file 42003_2023_5046_MOESM2_ESM.pdf]

Supplementary Information for

Nonclassical mechanisms to irreversibly suppress β-hematin crystal growth

Wenchuan Ma, Victoria A. Balta, Weichun Pan, David J. Sullivan, Jeffrey D. Rimer, Peter G. Vekilov

Supplementary Figures

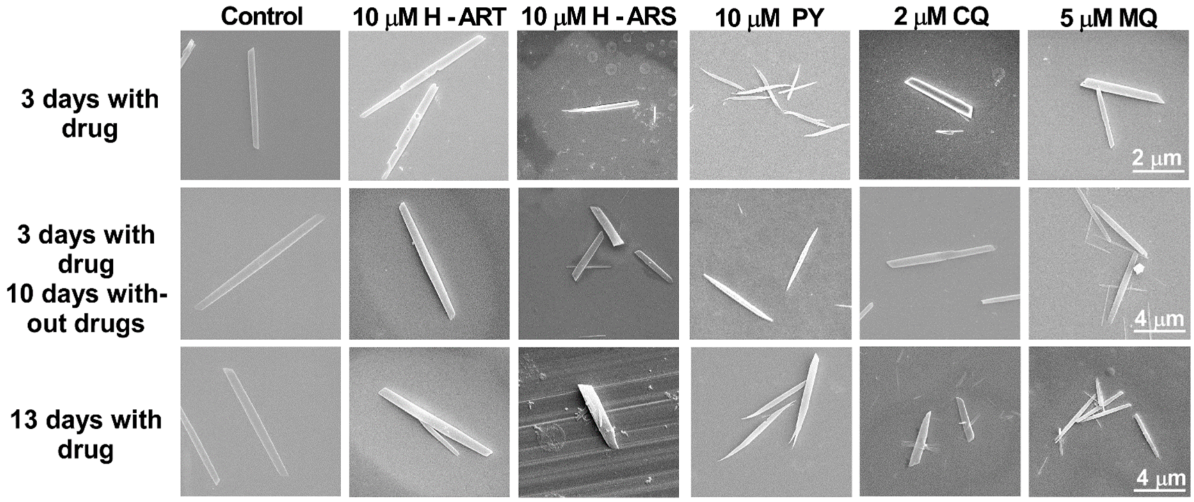

Supplementary Fig. 1. SEM micrographs of β-hematin crystals grown on the presence of H-ART, H-ARS, PY, CQ and MQ at concentrations indicated above the images in the three regimes illustrated in Fig. 1d.

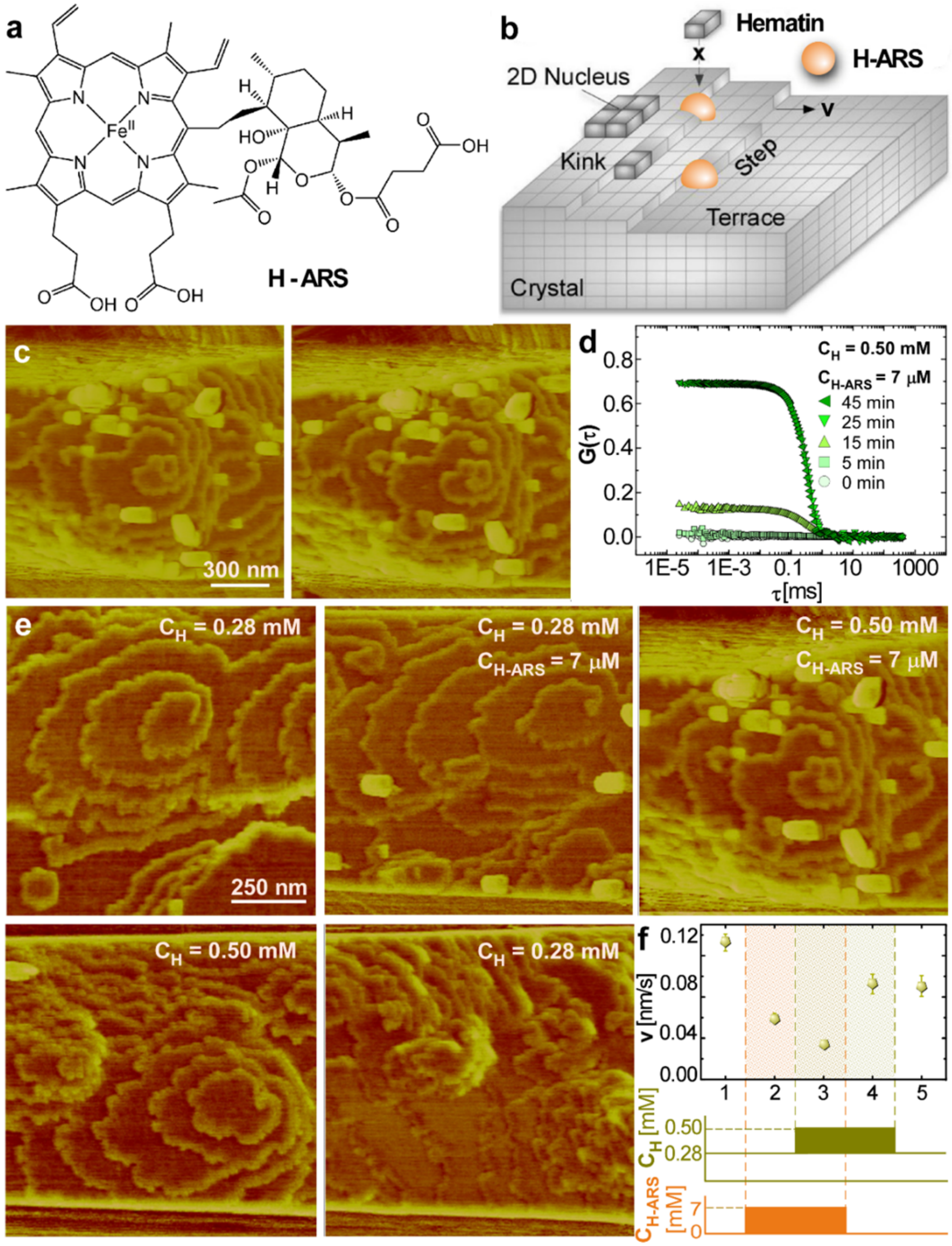

Supplementary Fig. 2. The molecular mechanism of irreversible inhibition of β-hematin crystallization by H-ARS. a, The structure of H-ARS. **b,** Schematic illustration of step inhibition by kink blockers, which associate to the kinks and obstruct the access of solute molecules. **c,** AFM images of (100) β-hematin crystal surfaces at C_H = 0.50 mM and 10 H-ARS. **d,** Evolution of the correlation functions $G(\tau)$ of scattered light over 40 min in solutions with C_H = 0.5 mM and 10 μM H-ARS. Deviations from zero manifest the formation of aggregates. **e,** Sequential AFM images of step patterns on the (100) β-hematin crystal surface at hematin and H-ART concentrations indicated on the panel. **f,** The step velocity v at different combinations of hematin and H-ART concentrations, corresponding to morphologies in **e**. Error bars denote the standard deviations of ca. 30 measurements.

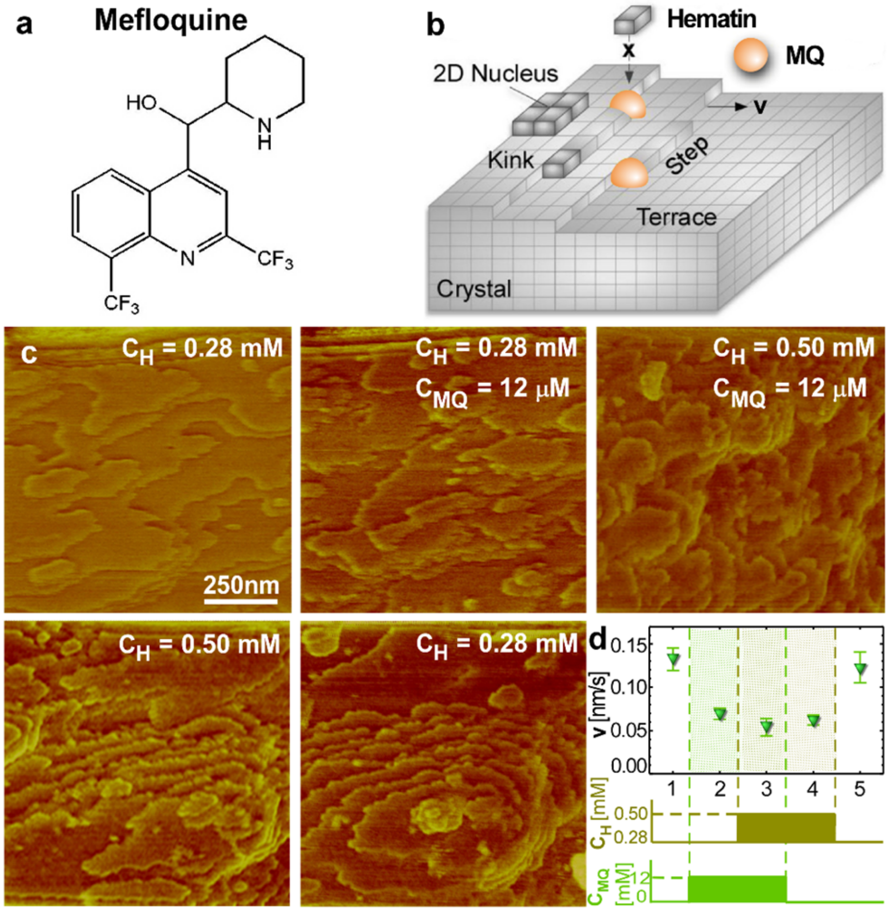

Supplementary Fig. 3. Reversible inhibition by MQ. **a,** The structure of MQ. **b,** Schematic illustration of step inhibition by kink blockers, which associate to the kinks and obstruct the access of solute molecules. **c,** Step patterns on the (100) β-hematin crystal surface at hematin and MQ concentrations indicated on the panels. **d,** The step velocity v at different combinations of hematin and MQ concentrations, corresponding to the morphologies in panel **c.**

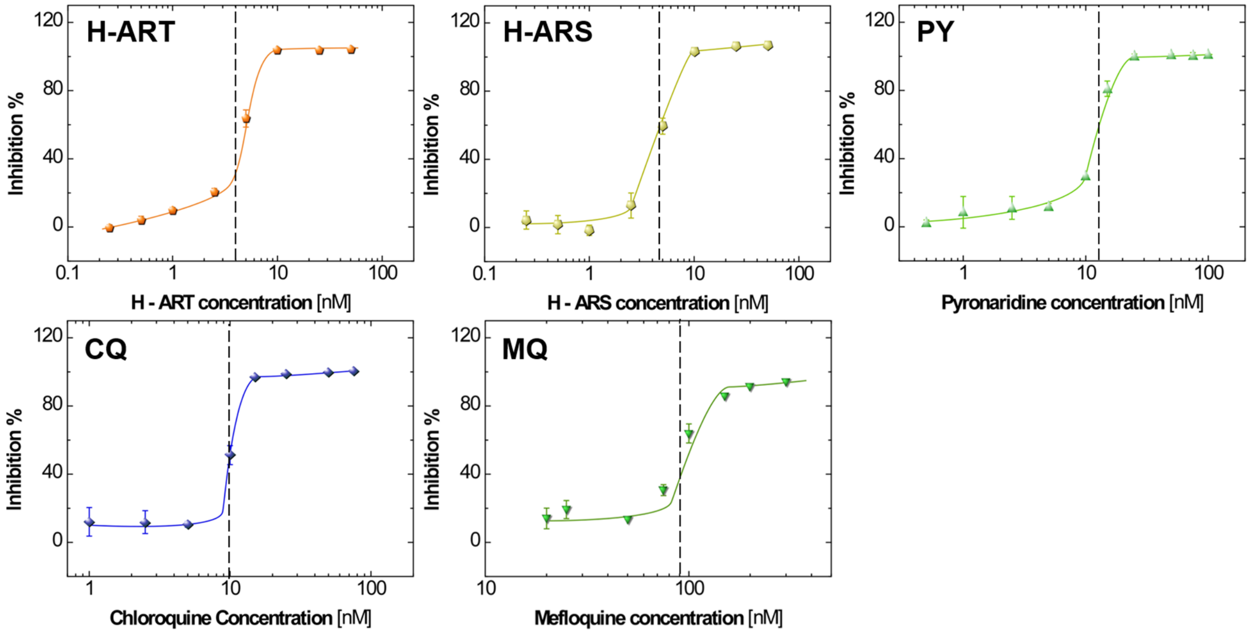

Supplementary Fig. 4. Percent inhibition of *P. falciparum* strains NF54 after 72 hours of continuous exposure to H-ART, H-ARS, PY, CQ or MQ as a function of inhibitor concentration. Vertical dashed lines denote the IC_{50} values, the concentration of a metabolite or drug that inhibits 50% of the parasites after 72-hour exposure, which are for H-ART, 4.0 nM; for H-ARS, 4.4 nM; for PY, 11.6 nM; for CQ, 9.8 nM; and for MQ; 88.2 nM.

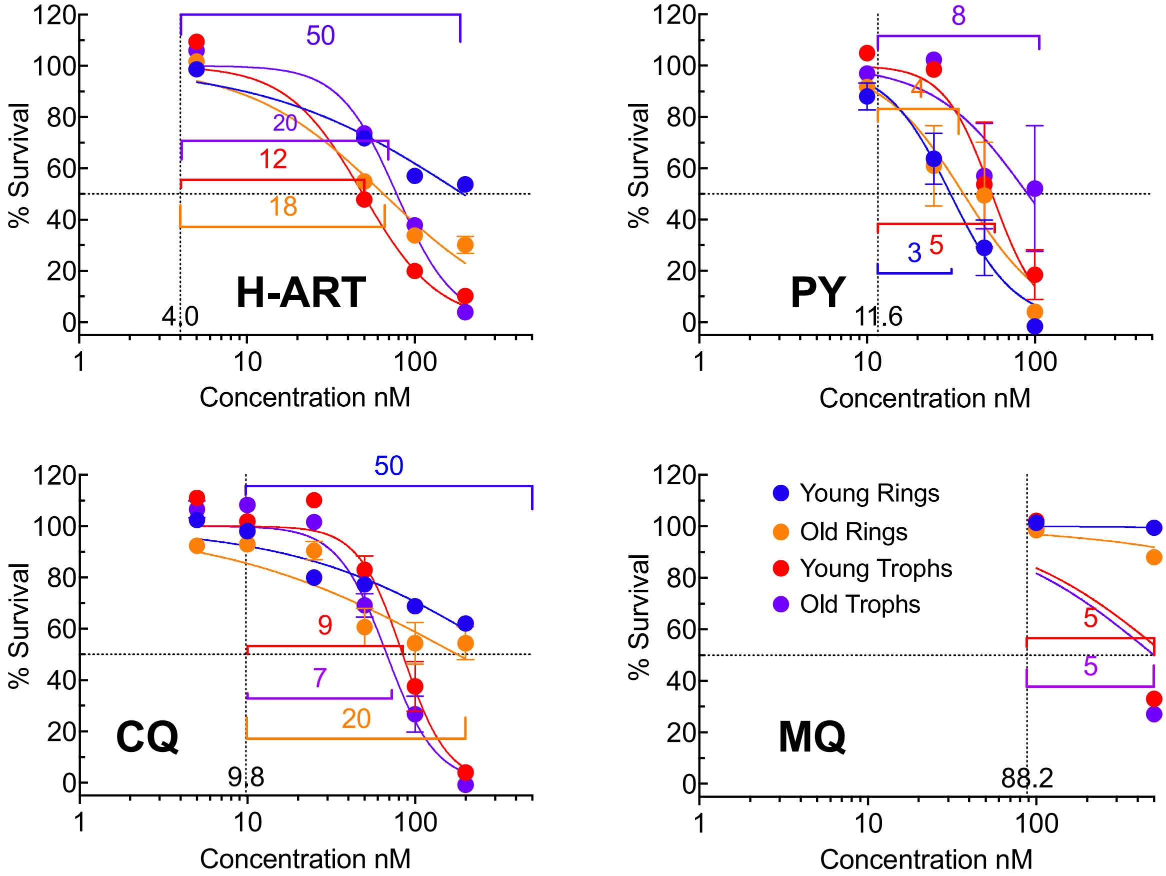

Supplementary Fig. 5. Fraction of parasites surviving for 72 hours after their lifecycles were synchronized. The parasites were exposed for three hours to H-ART, PY, CQ, and MQ, introduced at 0, 5, 24 and 29 hours of their lives. These parasite ages correspond to young ring-stage parasites, old ring-stage parasites, young trophozoites and old trophozoites, respectively. The legend is in the MQ panel. Vertical dotted lines and adjacent numbers denote independently measured continuous inhibitor IC_{50} values, the concentration of a metabolite or drug that inhibits 50% of the parasites with a 72-hour exposure. Horizontal dotted lines mark the 50% parasite survival. Horizontal brackets and adjacent numbers denote fold ratios of the inhibitor concentrations that suppress 50% of parasites of each age in a 3 hour pulse to the continuous inhibitor's IC_{50}

Supplementary Tables

Supplementary Table 1. The analysis of variance (ANOVA) p and F parameters for the length and width increment for the last 10 days as described in criteria 1 and 2 in the text. $p < 0.05$ and $F > F_{crit}$ indicate that the two analyzed distributions are distinct, i.e., the null hypothesis is false, whereas $p > 0.05$ and $F < F_{crit}$ indicate that the null hypothesis is true and two distributions are similar. Applying Criterion 1, true indicates reversible inhibition (R) and false, irreversible (IR), whereas for criterion 2, true indicates irreversible inhibition (IR) and false, reversible (R). Starred (*) Criterion 2 false outcomes for H-ARS and PY indicate faster growth of crystals in the presence of the additives than in their absence and irreversible inhibition

Length, {011} faces

	Criterion 1					Criterion 2				
	p	F	F_{crit}	True/ False	R/IR	p	F	F_{crit}	True/ False	R/IR
H-ART	0.55	0.35	4.01	True	R	2e-06	27	4.00	False	R
H-ARS	1e-16	134	4.01	False	IR	0.07	3.33	4.00	True	IR
PY	2e-07	35	4.01	False	IR	0.37	0.8	4.00	True	IR
CQ	0.03	5.12	4.03	False	IR	1e-17	162	4.00	False	R
MQ	0.05	8.55	4.01	False	IR	6e-08	38	4.00	False	R

Width, {010} faces

	Criterion 1					Criterion 2				
	p	F	F_{crit}	True/ False	R/IR	p	F	F_{crit}	True/ False	R/IR
H-ART	0.17	1.91	4.01	True	R	4e-11	66	4.01	False	R
H-ARS	3e-10	57	4.01	False	IR	0.002	10.5	4.01	False	IR*
PY	1e-11	71	4.01	False	IR	0.027	5.07	4.00	False	IR*
CQ	0.005	8.6	4.02	False	IR	0.21	1.6	4.02	True	IR
MQ	0.0007	13	4.03	False	IR	0.0002	15	4.00	False	R

Supplementary Table 2. IC50 concentrations for 3 and 6 hour pulses.

H-ART	6hr IC50	95% CI	72 hr ic50	ratio 6 hr	3hr IC50	95% CI	72 hr ic50	ratio 3 hr
Young Rings	37.6	33-43	4	9	194	147-290	4	48
Old Rings	21.3	17-26	4	5	65	51-81	4	16
Young Trophs	31.4	27-35	4	8	49	41-56	4	12
Old Trophs	35.2	31-39	4	9	78	72-85	4	20
H-ARS								
Young Rings	75	66-86	4.4	17			4.4	
Old Rings	46.5	37-58	4.4	11			4.4	
Young Trophs	47.8	40-56	4.4	11			4.4	
Old Trophs	43.3	39-48	4.4	10			4.4	
CQ								
Young Rings	76.8	68-87	9.8	8	350	277-478	9.8	36
Old Rings	58.4	50-68	9.8	6	178	107-491	9.8	18
Young Trophs	51.4	47-57	9.8	5	84	74-96	9.8	9
Old Trophs	54.7	48-62	9.8	6	68	61-76	9.8	7
MQ								
Young Rings	N/A				N/A			
Old Rings	N/A				N/A			
Young Trophs	194.6	170-222	88	2	592	316-1204	88	7
Old Trophs	177.1	152-206	88	2	499	254-1005	88	6
PY								
Young Rings	20	17-24	11.6	1.7	31	25-39	11.6	3
Old Rings	26.5	19-36	11.6	2.3	38	23-57	11.6	4
Young Trophs	56.4	48-64	11.6	5	55	41-79	11.6	5
Old Trophs	100.8	85-139	11.6	9	91	50-5670	11.6	8
