# Supplementary material for: Nonclassical mechanisms to irreversibly suppress β-hematin crystal growth
Source: Commun Biol. 2023 Jul 27;6:783. doi: 10.1038/s42003-023-05046-z (PMC10374632; doi:10.1038/s42003-023-05046-z)
Supplement: Supplementary file 3 — Description of Additional Supplementary Files [file 42003_2023_5046_MOESM3_ESM.pdf]

Description of Additional Supplementary Files

File name: Supplementary Data 1
Description: Data for all plots in Fig 1 and ANOVA analyses of comparisons between length and width increments.

File name: Supplementary Data 2
Description: Data for all plots in Fig. 2.

File name: Supplementary Data 3
Description: Data for all plots in Fig. 3.

File name: Supplementary Data 4
Description: Data for all plots in Fig. 4.

File name: Supplementary Data 5
Description: Data for all plots in Fig. 5
